# Identification of a Novel Antimicrobial Peptide from Human Hepatitis B Virus Core Protein Arginine-Rich Domain (ARD)

**DOI:** 10.1371/journal.ppat.1003425

**Published:** 2013-06-13

**Authors:** Heng-Li Chen, Pei-Yi Su, Ya-Shu Chang, Szu-Yao Wu, You-Di Liao, Hui-Ming Yu, Tsai-Ling Lauderdale, Kaichih Chang, Chiaho Shih

**Affiliations:** 1 Institute of Biomedical Sciences, Academia Sinica, Taipei, Taiwan; 2 Taiwan International Graduate Program in Molecular Medicine, National Yang-Ming University and Academia Sinica, Taipei, Taiwan; 3 Graduate Institute of Microbiology and Immunology, National Yang-Ming University, Taipei, Taiwan; 4 Genomics Research center, Academia Sinica, Taipei, Taiwan; 5 Microbial Infections Reference Laboratory (MIRL), National Health Research Institute (NHRI), Zhunan Town, Taiwan; 6 Department of Laboratory Medicine and Biotechnology, Tzu Chi University, Hualien City, Taiwan; University of California, San Diego, United States of America

## Abstract

The rise of multidrug-resistant (MDR) pathogens causes an increasing challenge to public health. Antimicrobial peptides are considered a possible solution to this problem. HBV core protein (HBc) contains an arginine-rich domain (ARD) at its C-terminus, which consists of 16 arginine residues separated into four clusters (ARD I to IV). In this study, we demonstrated that the peptide containing the full-length ARD I–IV (HBc147-183) has a broad-spectrum antimicrobial activity at micro-molar concentrations, including some MDR and colistin (polymyxin E)-resistant *Acinetobacter baumannii*. Furthermore, confocal fluorescence microscopy and SYTOX Green uptake assay indicated that this peptide killed Gram-negative and Gram-positive bacteria by membrane permeabilization or DNA binding. In addition, peptide ARD II–IV (HBc153-176) and ARD I–III (HBc147-167) were found to be necessary and sufficient for the activity against *P. aeruginosa* and *K. peumoniae*. The antimicrobial activity of HBc ARD peptides can be attenuated by the addition of LPS. HBc ARD peptide was shown to be capable of direct binding to the Lipid A of lipopolysaccharide (LPS) in several *in vitro* binding assays. Peptide ARD I–IV (HBc147-183) had no detectable cytotoxicity in various tissue culture systems and a mouse animal model. In the mouse model by intraperitoneal (i.p.) inoculation with *Staphylococcus aureus*, timely treatment by i.p. injection with ARD peptide resulted in 100-fold reduction of bacteria load in blood, liver and spleen, as well as 100% protection of inoculated animals from death. If peptide was injected when bacterial load in the blood reached its peak, the protection rate dropped to 40%. Similar results were observed in *K. peumoniae* using an IVIS imaging system. The finding of anti-microbial HBc ARD is discussed in the context of commensal gut microbiota, development of intrahepatic anti-viral immunity and establishment of chronic infection with HBV. Our current results suggested that HBc ARD could be a new promising antimicrobial peptide.

## Author Summary

Antibiotics-resistant pathogens have been a major problem to our public health. Recently, in our studies of human hepatitis B virus (HBV), we accidentally discovered potent and broad spectrum antimicrobial peptides from HBV core protein (HBc) arginine-rich domain (ARD). The peptides are mainly composed of SPRRR repeats and are effective against both Gram-positive and Gram-negative bacteria, as well as fungi. We found different bactericidal mechanisms of the ARD peptides, which involved LPS binding, DNA binding and membrane permeabilization in various tested bacteria, such as *P. aeruginosa*, *K. pneumoniae*, *E. coli* and *S. aureus*. We also found that this ARD peptide was effective for colistin-resistant *A. baumannii*. The peptides exhibited no hemolysis activity to human red blood cells and no cytotoxicity to human hepatoma cells and kidney cells. Furthermore, the ARD peptide was shown to be safe and protective in the animal model. Recently, intestinal flora was found to influence the development of immunity. We discussed here the potential involvement of the antimicrobial activity of HBc ARD in the establishment of HBV chronic infection in the newborns. We proposed here that the HBc ARD peptides could serve as an alternative to the conventional antibiotics in clinical medicine.

## Introduction

The increase of drug-resistant pathogens caused by the extensive use of traditional antibiotics is a serious concern worldwide. There is an urgent need to develop more effective treatment to overcome the drug-resistance problem. Antimicrobial peptides (AMP) are a new class of antibiotics with a new mode of action and remarkable therapeutic effects [1]. In general, they contain 10–50 amino acids, with an overall positive charge and an amphipathic structure. Under hydrophobic environment, AMPs can fold into four classes of structures, including α-helix, β-sheets, extended structures, and loops [1], [2], [3]. It is well known that most AMPs can directly bind to bacteria membrane and kill them by disrupting membrane or targeting intracellular components [3], [4], [5]. Most importantly, they are effective to antibiotics-resistant pathogens [6], [7]. This unique feature has encouraged the development of AMPs as novel antibiotics in the last few decades. To date, more than one thousand AMPs have been identified in various species including plants, insects, fish, frogs, and mammals [8], [9], [10], [11], [12], [13]. Although their sequences vary greatly, certain amino acids such as cysteine, lysine, proline or arginine are key compositions of AMPs [12], [14], [15], [16], [17].

Hepatitis B virus (HBV) remains a major human pathogen, and there are new challenges for the treatment of viral hepatitis B [18], [19]. HBV encodes a 21 KDa core (HBc) protein, which is essential for viral replication [20], [21], [22]. It contains a capsid assembly domain at N-terminus (residue 1 to 149) and an arginine-rich domain (ARD) at C-terminus (residues 150 to 183) [23], [24]. ARD contains 16 arginines separated into four arginine-rich clusters (ARD I, II, III, IV) and has a function of binding to nucleic acids. When it binds to HBV pre-genomic RNA or polyanions, HBc can assemble into a stable capsid [25], [26], [27]. In addition, ARD contains important signals for nuclear export and import of HBc core protein and particles [28]. We have found that the growth of *E. coli* expressing HBc1-183 was much slower than that of *E. coli* expressing HBc1-149 (unpublished results). It appears that it is HBc 150-183 that somehow retarded the growth of *E. coli*, and dramatically reduced the yield of HBc 1-183 protein. When we examined the sequences of HBc 150-183 in further detail, we noted that it shares a certain degree of sequence similarity with known antimicrobial peptides [29], [30]. This finding suggests the possibility that ARD may have the antimicrobial activity. In this study, we determined the *in vitro* antimicrobial activities of HBc147-183 against a wide variety of bacteria, including multidrug resistant (MDR) and colistin (polymyxin E)-resistant *A baumannii*. Using a peritoneal sepsis mouse model, we demonstrated further that ARD peptides can effectively protect all the mice challenged with a lethal dose of *Staphylococcus aureus*. Treatment of ARD peptide also caused significant reduction of bacterial load of *S. aureus* and *K. pneumoniae* in infected mice. Potential mechanisms for the bactericidal activity were investigated. The ARD peptides appeared to be capable of direct binding to the Lipid A moiety of lipopolysaccharide (LPS) in several different binding assays. We discussed further the potential significance of the anti-microbial activity of the HBc ARD peptide in the commensal microbiota and the development of the intrahepatic antiviral immunity in HBV infected newborns. In summary, with high antimicrobial activity and very low toxicity against human cells and animal models, these HBc ARD peptides may have a therapeutic potential in the future.

## Materials and Methods

### Ethics statement

All animal experiments were conducted under protocols approved by Academia Sinica Institutional Animal Care & Utilization Committee (ASIACUC permit number 12-02-322). Research was conducted in compliance with the principles stated in the Guide for the Care and Use of Laboratory Animals, National Research Council, 1996.

### Bacterial isolates

The antimicrobial activities of HBc ARD peptides were tested using a number of bacterial strains from ATCC, including *Pseudomonas aeruginosa* Migula strain (ATCC 27853, ampicillin-resistant), *Pseudomonas aeruginosa* Migula strain (ATCC 9027, ampicillin-resistant), *Klebsiella pneumoniae* strain (ATCC 17593), *Escherichia coli* strain (ATCC 25922), *Staphylococcus aureus* subsp. strain (ATCC 25923, methicillin-resistant), *Staphylococcus aureus* subsp. strain (ATCC 29213, methicillin-resistant), *Staphylococcus aureus* subsp. strain (ATCC 19636, methicillinresistant), and *Candida albicans* strain (ATCC 10231).

Clinical isolates of *Pseudomonas aeruginosa* (NHRI-01, NHRI-02 and NHRI-04) were obtained through the program of Taiwan Surveillance of Antimicrobial Resistance (TSAR), National Health Research Institutes (NHRI), Taiwan. *Acinetobacter baumannii* (ATCC 17989, ATCC 17978 CR, ATCC19606, ATCC 19606 CR, TCGH 45530 and TCGH 46709) were obtained from Tzu-Chi Buddhist General Hospital (TCGH) in Taiwan, and clinical isolates (TCGH 45530 and TCGH 46709) were identified using the Vitek system (Biomerieux Vitek, Inc., Hazelwood, MO, USA) [31]. A. baumannii is defined as multidrug-resistant, when the organism is resistant to piperacillin, piperacillin-tazobactam, ampicillin/sulbactam, imipenem, ceftazidime, gentamicin, amikacin, tetracycline, chloramphenicol, ciprofloxacin, and cotrimoxazole [32]. Susceptibility to colistin was determined using the broth-dilution method, in accordance with the guidelines of the Clinical and Laboratory Standards Institute (CLSI) [33].

### Antimicrobial activity

All peptides were purchased from Yao-Hong Biotechnology Inc. (Taipei, Taiwan). Vendors provided data of peptide characterizations, including HPLC and Mass (data not shown). Antimicrobial activity was determined as described [30] with some modifications as detailed below. Bacteria were grown overnight in Mueller–Hinton broth (Difco) at 37°C, and during the mid-logarithmic phase, bacteria were diluted to 10^6^ CFU (colony formation unit)/ml in phosphate buffer (10 mM sodium phosphate and 50 mM sodium chloride, pH 7.2). Peptides were serially diluted in the same buffer. Fifty microliter (µl) of bacteria was mixed with fifty µl of peptides at varying concentrations followed by incubation at 37°C for 3 hours without shaking. At the end of incubation, bacteria were placed on Mueller-Hinton broth agar plates, and allowed growth at 37°C overnight for measurement of minimal bactericidal concentration (MBC). The lowest peptide concentration on the agar plate, which displayed no bacterial growth (zero colony), is defined as MBC. All peptides were tested in triplicate.

For measurement of killing kinetics, bacteria and peptides were prepared as described above. Fifty µl of bacteria were mixed with fifty µl of peptides at the concentrations corresponding to MBC and were incubated at 37°C. At the indicated time, bacteria were serially diluted and placed on Mueller-Hinton broth agar plates for the viability measurement.

### Confocal fluorescence microscopy

The localization of peptide was monitored by confocal fluorescence microscopy. Bacteria were grown to mid-logarithmic phase and were collected by centrifugation. Approximate 10^7^ CFU were resuspended in phosphate buffer containing FITC-labeled HBc147-183 at a concentration corresponding to 0.5×MBC. Following incubation for 1 hour at 37°C, cells were washed, fixed, and immobilized on poly-L-lysine coated glass slides. ProLong Gold antifade reagent with DAPI (Invitrogen) was added to the slides prior to mounting. Localization of labeled-peptide was observed using an Olympus Ultraview confocal microscopy equipped with a 100× oil immersion lens.

### SYTOX Green uptake

Briefly, bacteria (10^7^ CFU) were prepared and mixed with 1 µM SYTOX Green (Invitrogen) for 15 minutes in the dark. After the addition of peptides to the final concentrations corresponding to their respective MBC, fluorescence intensity was measured at 37°C using wavelengths 485 nm and 520 nm filters for excitation and emission. Melittin (Sigma), the major toxin of bee venom, was used as a positive control to provide maximal permeabilization [34].

### Gel retardation assay

The proportion between amino nitrogen (NH_3_^+^) of HBc147-183 and phosphate (PO_4_^−^) of DNA was defined as N/P ratio [35]. Briefly, HBc147-183 was incubated with pSUPER plasmid DNA at different N/P ratio (0, 0.2, 0.4, 0.6, 0.8, 1, 2, 3 and 4) for 30 minutes at 37°C. The mobility of pSUPER plasmid DNA was analyzed by electrophoresis on 1% agarose gel.

### *In vitro* binding assay between ARD peptides and LPS/LipidA

Several kinds of peptide-LPS or peptide-Lipid A binding assays were performed in this study: 1) Streptavidine-conjugated beads (Dynabeads MyOne Streptavidin T1, Invitrogen) were blocked by *P. aeruginosa* LPS (Sigma) at 37°C for 1.5 hour. After washing with PBST (PBS, pH 7.4 containing 0.1% (w/v) Tween-20), aliquots containing 250 pmol streptavidine-conjugated beads were incubated with a reaction mixture overnight at 4°C. The reaction mixture was prepared by mixing increasing amounts of biotinylated peptide HBc147-183 (0, 0.004, 0.02, 0.1, 0.5 and 2.5 µM) and 5 µg/ml *P. aeruginosa* LPS (Sigma) or 200 µg/ml *E. coli* lipid A (Sigma), at 37°C for 3 hour. After incubation overnight at 4°C, the reduction of LPS (or Lipid A) in the supernatants were measured by the Limulus Amebocyte Lysate (LAL) test (Charles River Endosafe) with an ELISA reader (Molecular Devices). The amount (EU/ml) of LPS was calculated according to the standard curve prepared with Endosafe Control Srandard Endotoxin. 2) To directly measure the increasing amounts of LPS bound to the increasing amounts of peptide HBc147-183 on the peptide-coated beads, the beads were then washed with PBST three times and incubated with 100 µl of PBS containing 0.15 units of trypsin agarose (Sigma) for overnight digestion at 37°C. After trypsinization, the trypsin-released LPS in the supernatants were collected and measured by the Limulus Amebocyte Lysate (LAL) test. Similar results were obtained by another LPS testing method: Endosafe- PST Cartridges (Charles River Laboratories). 3) To perform the LPS/Lipid A competition assay, one µg of LPS was coated on High Binding ELISA plates (Corning) overnight at 4°C. The LPS-coated plates were washed by PBST and then blocked with PBST containing 5% BSA for 1 hour at 37°C. After washing, HBc147-183 (10 nM), mixed with varying concentrations of *E. coli* Lipid A (0 to 10 µg/ml), were added into each well and incubated for 1 hour at 37°C. Plate-bound HBc147-183 was measured by streptavidin conjugated with HRP (1∶10000 dilution) for 1 hour at 37°C. TMB substrates were added into each well for color development. The absorption was measured at 450 nm with a reference wavelength at 655 nm.

### Hemolytic activity

The hemolytic activities of peptides were determined by hemolysis against human red blood cells (hRBCs). Human blood was obtained in EDTA-containing tube and was centrifuged at 450 g for 10 min. The pellet was washed three times with PBS buffer, and a solution of 10% hRBCs was prepared. hRBCs solution was mixed with serial dilutions of peptides in PBS buffer, and the reaction mixtures were incubated for 1 h at 37°C. After centrifugation at 450 g for 10 min, the percentage of hemolysis was determined by measuring the absorbance at the wavelength of 405 nm of the supernatant. Blank and 100% hemolysis were determined in PBS buffer and in the presence of 1% Triton X-100, respectively.

### Cytotoxicity

Cytotoxicity was measured for HepG2, Huh7, HEK293, and Vero cells by MTT assay. Cells were seeded at 10^4^ cells/well in a 96-well plate and serial dilutions of peptides were added into each well. PBS was used as a negative control and melittin was used as a positive control. After 1 hour of incubation, the medium were replaced by fresh medium containing 10% MTT solution (Promega), and the plate was incubated for 4 hours in 5% CO_2_ at 37°C. The absorbance at the wavelength of 595 nm was measured by an ELISA reader (Bio-Rad model 680).

### CFSE cell proliferation assay

To set up CFSE cell proliferation assay, 293 cells (human kidney origin) and Vero cells (monkey kidney origin) were resuspended in PBS to a final concentration of 10^6^ cells/ml, before incubation with 10 µM CFSE dye (CellTrace CFSE cell proliferation kit, Invitrogen) at 37°C for 10 min. To quench the staining, ice-old culture media were then added and incubated on ice for 5 min. Labeled cells were then pelleted and washed three times by fresh medium containing 10% FBS before seeding into six well plates at a density of 3.3×10^5^cells/well. After 20 h, the medium was removed and incubated with fresh medium containing 5, 25 and 100 µM HBc 147-183 for one hour (FITC-labeled ARD peptide had been largely internalized in 10 minutes after the addition of ARD peptides to the medium of HepG2 cells). Forty-eight hours later, cells were harvested and analyzed by flow cytometry (FACSCanto, BD Bioscience).

### *In vivo* animal studies

Three-week old male ICR mice (19 to 21 g) were purchased from BioLASCO (Taiwan). Overnight culture of bacteria in BHI broth (Difco) was subcultured in fresh BHI broth to log phase. Inoculums were diluted in BHI broth to indicated densities. To test the acute toxicity of ARD peptide *in vivo*, ICR male mice were inoculated intraperitoneally (i.p.) with 10 and 20 mg/kg HBc147-183 in PBS, respectively. Each group contained 5 mice. After peptide injection, the number of dead mice was recorded daily for 7 days post-injection. To test the antimicrobial activity of the ARD peptide *in vivo*, all mice were inoculated i.p. with *Staphylococcus aureus* ATCC 19636 (4×10^6^ CFU/mouse) in BHI broth. Peptide HBc147-183 (10 mg/kg) was administered i.p. at 1, 1.5 and 2 hours post-inoculation. PBS (10 ml/kg) control was administered at 1 hour post-inoculation. Each group contained 10 mice. Mortality was monitored daily for 7 days post-inoculation. In a separate experiment to measure the bacterial load, mice were inoculated i.p. with *Staphylococcus aureus* ATCC 19636 (10^6^ CFU/mouse) in BHI broth. All mice were administered at 1 hour post-inoculation with peptide HBc147-183 (10 mg/kg) or PBS (10 ml/kg) control, and sacrificed at 4 hours post-inoculation. Blood samples (200 µl) were mixed with 100 mM EDTA (10 µl) and were diluted 20-fold in PBS (calcium and magnesium free). Liver and spleen samples (0.1 g) were homogenized in sterile PBS (500 µl). Samples were diluted approximately 100-fold and plated on BHI agar for scoring the colony numbers. To test the *in vivo* antimicrobial activity of the ARD peptide against Gram-negative bacteria, mice were inoculated with *Klebsiella pneumoniae* Xen39 (10^7^ cfu/mouse) (Caliper LifeSciences), an engineered strain containing a modified *Photorhabdus luminescens luxABCDE* operon. One hour post-inoculation, mice received either 10 ml/kg PBS (n = 5) or 10 mg/kg ARD peptide (n = 5), respectively. *In vivo* imaging was carried out at 4 hours post-inoculation. The mice were anesthetized first before transferring to the IVIS imaging system (IVIS spectrum), and luminescence was measured with an exposure time of 1 minutes or less. The image system measured the number of photons and translated the data to false color images that depicted the region of strong luminescence with red, moderate luminescence with yellow and green, and mild luminescence with blue. Decreasing bioluminescence indicated reduction of bacteria. The images were overlay of photographic images and bioluminescence using a computer-generated color scale. Total flux (RLU) of region of interest (ROI) was quantified by the IVIS imaging software.

## Results

### *In vitro* antimicrobial activity of HBc peptides

**Figure 1:**
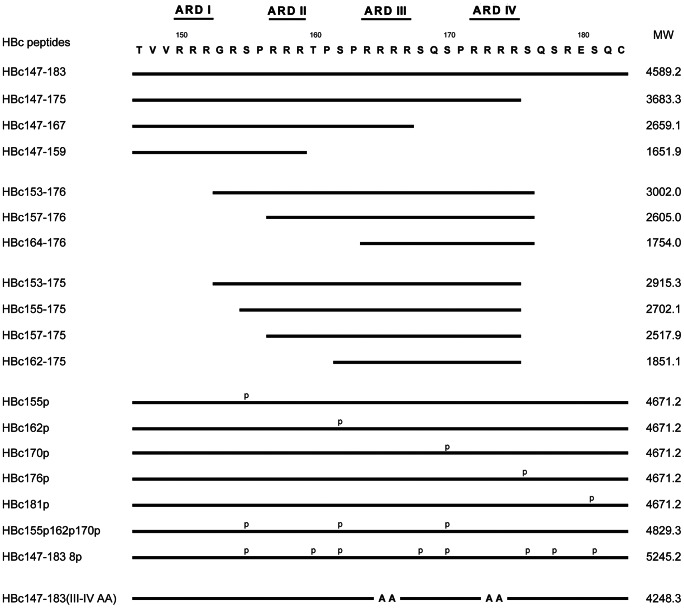
Amino acid sequences of various HBc ARD peptides tested for bactericidal activity in this study. The lower panel presents various phosphorylated peptides and an R-to-A mutant peptide with a total of four arg-to-ala substitutions in ARD-III and ARD-IV of HBc147-183.

As shown in Figure 1 and Table 1, HBc147-183 displayed a broad-spectrum activity against Gram-negative bacteria (*P. aeruginosa*, *K. pneumoniae* and *E. coli*), Gram-positive bacteria (*S. aureus*), and fungi (*C. albicans*). Among these tested strains, *P. aeruginosa* and *K. pneumonia* were the most sensitive to this peptide. The MBCs of HBc147-183 were lower than 4 µM for *P. aeruginosa* and *K. pneumonia*, and around 4 µM for *E. coli*, and *S. aureus*. *C. albicans* was the least sensitive to this peptide (MBC ∼8 µM).

**Table 1:** Antimicrobial activity of HBC ARD peptides.

Peptide	Antimicrobial activity^a^ µM (µg/ml)							
	Gram-negative bacteria				Gram-positive bacteria			Fungi
	*P. aeruginosa* (ATCC 9027)^b^	*P. aeruginosa* (ATCC 27853)^b^	*K. pneumonia* (ATCC 13884)	*E. coli* (ATCC 25922)	*S. aureus* (ATCC 19636)^c^	*S. aureus* (ATCC 25923)^c^	*S. aureus* (ATCC 29213)^c^	*C. albicans* (ATCC 10231)
HBc147-183	2 (9.18)	2–4 (9.18–18.36)	2 (9.18)	4 (18.36)	4 (18.36)	4 (18.36)	4 (18.36)	∼8 (∼36.7)
HBc147-175	0.5 (1.84)	1 (3.68)	1 (3.68)	1 (3.68)	>32 (>117.86)	>32 (>117.86)	>32 (>117.86)	∼8 (∼36.7)
HBc147-167	>32 (>85)	>32 (>85)	8 (21.3)	>32 (>85)	ND	ND	ND	ND
HBc147-159	ND	ND	ND	ND	ND	ND	ND	ND
HBc153-176	1–2 (3–6)	1–2 (3–6)	>32 (>96)	16 (48)	ND	ND	ND	ND
HBc157-176	2 (5.2)	2 (5.2)	>32 (>83.36)	>32 (>83.36)	ND	ND	ND	ND
HBc164-176	ND	ND	ND	ND	ND	ND	ND	ND
HBc153-175	2 (5.8)	2 (5.8)	>32 (93.28)	16 (46.64)	ND	ND	ND	ND
HBc155-175	2 (5.4)	16 (43.23)	>32 (>86.46)	>32 (>86.46)	ND	ND	ND	ND
HBc157-175	2 (10)	>32 (>80.58)	>32 (>80.58)	>32 (>80.58)	ND	ND	ND	ND
HBc162-175	ND	ND	ND	ND	ND	ND	ND	ND
HBc155p	8 (37.4)	8 (37.4)	1 (4.67)	8 (37.4)	8 (37.4)	8 (37.4)	8 (37.4)	ND
HBc162p	∼32 (∼149.5)	∼32 (∼149.5)	∼32 (∼149.5)	∼32 (∼149.5)	∼32 (∼149.5)	∼32 (∼149.5)	∼32 (∼149.5)	ND
HBc170p	∼32 (∼149.5)	∼32 (∼149.5)	8 (37.4)	∼32 (∼149.5)	∼32 (∼149.5)	∼32 (∼149.5)	∼32 (∼149.5)	ND
HBc176p	8 (37.4)	8 (37.4)	4 (18.68)	8 (37.4)	8 (37.4)	8 (37.4)	8 (37.4)	ND
HBc181p	2 (9.3)	4 (18.68)	4 (18.68)	4 (18.68)	4 (18.68)	4 (18.68)	4 (18.68)	ND
HBc155p162p170p	>32 (>154.5)	>32 (>154.5)	>32 (>154.5)	>32 (>154.5)	>32 (>154.5)	>32 (>154.5)	>32 (>154.5)	ND
HBc147-183(34AA)	32 (135.94)	32 (135.94)	32 (135.94)	>32 (>135.94)	>32 (>135.94)	>32 (>135.94)	>32 (>135.94)	ND
Melittin	2 (5.7)	2 (5.7)	2 (5.7)	2 (5.7)	1 (2.85)	2 (5.7)	1 (2.85)	4 (11.4)

a Antimicrobial activity were measured after incubation with peptides for 3 hours. Numbers here represent MBC (minimal bactericidal concentration). ND, not detectable.

b Ampicilin-resistant *Pseudomonas aeruginosa* Migula strain.

c Methicillin-resistant *Staphylococcus aureus* subsp. strain.

To further map the active sequences of the antimicrobial activity, various peptides (Figure 1) in different length were synthesized and tested as before. Peptide HBc147-175, with the deletion of the last eight amino acids at the C-terminus, maintained strong activity against Gram-negative bacteria, albeit it lost the activity against *S. aureus* and *C. albicans*. We detected no activity against all of the tested bacteria and fungi from peptides ARD I–II (HBc147-159) or ARD III–IV (HBc164-176 and HBc162-175). In contrast, all peptides containing ARD II–IV (HBc153-176, HBc157-176, HBc153-175, HBc155-175, and HBc157-175) and ARD I–III (HBc147-167) exhibited strong activity against *P. aeruginosa* and *K. pneumonia*, respectively, albeit they were weak against *E. coli* (Table 1). Therefore, peptide ARD II–IV and ARD I–III appeared to be necessary and sufficient for the bactericidal activity against *P. aeruginosa* and *K. pneumonia*.

### Positive charge of ARD peptides is critical to the bactericidal activity

Our phophorylation studies on serine residues S155, S162, S170, S176 and S181 revealed that serine phosphorylation in general weakened the potency of antimicrobial activity. For example, as shown in Table 1, we found that all HBc peptides, once phosphorylated, lost their activities against *C. albicans*. For bacteria, the phosphorylation on S181 showed no effects, whereas phosphorylations on S155, S162, S170, and S176 reduced the antimicrobial activity. The MBCs dropped to 8 µM for HBc155p and HBc176p, and 32 µM for HBc162p and HBc170p, respectively. When S155, S162 and S170 were simultaneously phosphorylated (HBc155p162p170p), the antimicrobial activity was completely lost (>32 µM). The results here suggested that, except for S181, serine phosphorylation is generally detrimental to the antimicrobial activity of HBc ARD peptide. To confirm the importance of arginine residues for bactericidal activity, we synthesized and tested peptide HBc147-183-III–IV AA, which has two R-to-A substitution mutations in each of ARD III and ARD IV. Similar to phosphorylated HBc ARD peptides, the MBC of HBc147-183-III–IV AA was significantly increased compared to HBc147-183. The result here indicated that arginine residues are required for the antimicrobial activity.

### Drug resistance

Cationic peptides, such as polymyxin B and E (colistin), have become one of the last options for multidrug resistant bacteria these days [36]. We therefore tested the antimicrobial activity of HBc147-183 against colistin-resistant *P. aeruginosa* and *A. baumannii*. As shown in Table 2, while HBc147-183 killed colistin-sensitive *P. aeruginosa* at 4 µM, colistin-resistant *P. aeruginosa* were cross-resistant to HBc147-183 (MBC>16 µM). In contrast to *P. aeruginosa*, the MBCs of HBc147-183 against colistin-sensitive and colistin-resistant *A. baumannii* are in a similar range of 0.5–1 µM. This result indicates that, for colistin-resistant *A. baumannii*, there is no cross-resistance to our ARD peptide HBc147-183.

**Table 2:** Antimicrobial activity of ARD peptide HBc147-183 against colistin-resistant and sensitive *P. aeruginosa* and *A. baumannii*.

*Bacteria Strains	#Description	MBC		
		#Colistin (µg/ml)	#Polymyxin B (µg/ml)	HBc147-183 (µg/ml)
*P. aeruginosa* NHRI-01	Colistin resistant strain	>8	4	>73.4
*P. aeruginosa* NHRI-02	Colistin sensitive strain	4	2	18.36
*P. aeruginosa* NHRI-04	Colistin sensitive strain	4	2	18.36
*A. baumannii* ATCC17978	Reference strain	4	4	2.30
*A. baumannii* ATCC17978 CR	Induced Colistin resistant strain	>8	8	2.30
*A. baumannii* ATCC19606	Reference strain	2	2	2.30
*A. baumannii* ATCC19606 CR	Induced Colistin resistant strain	8	>8	2.30
*A. baumannii* TCGH45530	Clinical isolate, multidrug resistance	>8	8	2.30
*A. baumannii* TCGH46709	Clinical isolate, multidrug resistance	>8	>8	4.59

* Colistin-sensitive and -resistant *P. aeruginosa* were obtained from NHRI, Taiwan, and colistin-sensitive and –resistant *A. baumannii* were obtained Tzu-Chi Buddhist.

General Hospital, Taiwan (31; Materials and Methods).

# The breakpoints of colistin and polymyxin B resistance are according to Clinical and Laboratory Standards Institute (CLSI) (2009).

### Killing kinetics

**Figure 2:**
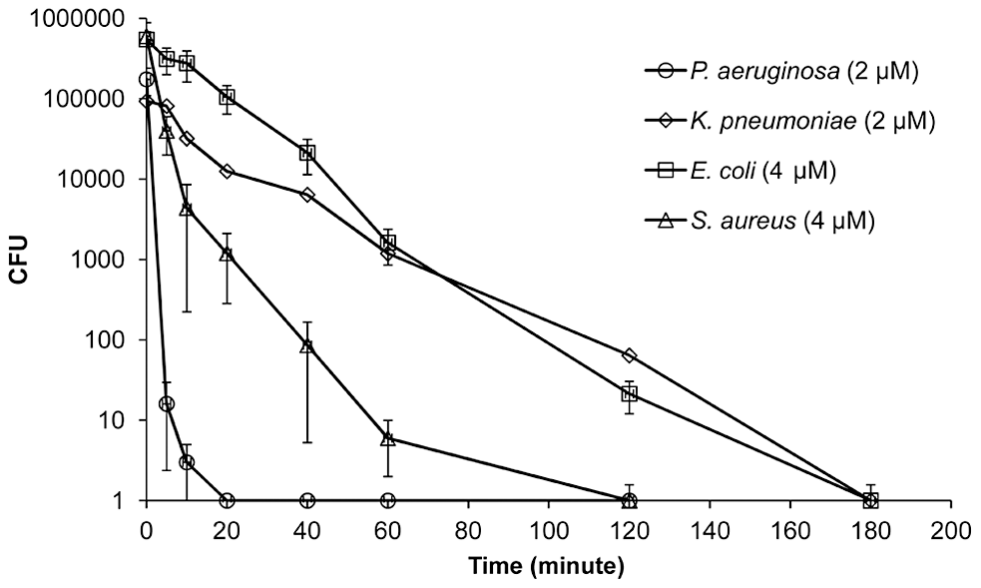
The killing kinetics of HBc147-183 against *P. aeruginosa* (circle), *K. pneumoniae* (diamond), *E. coli* (square) and *S. aureus* (triangle). Bacteria were treated with HBc147-183 (1×MBC). The viability of bacteria was measured at indicated time point. Samples were measured in triplicates.

Time course of bacterial viability was determined after the tested bacteria (*P. aeruginosa*, *K. pneumonia*, *E. coli* and *S. aureus*) were treated with HBc147-183 at the concentrations corresponding to the MBC (Figure 2). The results showed that *P. aeruginosa* was immediately killed within 20 minutes upon the addition of HBc147-183 (2 µM). Although *K. pneumonia* and *E. coli* were members of Gram-negative bacteria, they were killed by 4 µM HBc147-183 in 180 minutes. For *S. aureus*, complete killing by 4 µM HBc147-183 was observed in 120 minutes.

### Localization and mechanism of HBc147-183

**Figure 3:**
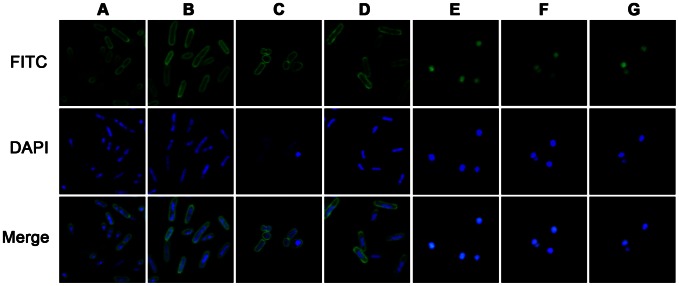
Localization of FITC-HBc147-183 peptide on the bacteria. Approximate 10^7^ CFU of *P. aeruginosa* ATCC9027, ATCC27853 (A and B), *K. pneumoniae* ATCC13884 (C), *E. coli* ATCC25922 (D), and *S.aureus* ATCC19636, ATCC25923 and ATCC 29213 (E, F and G) were incubation with HBc147-183 (0.5×MBC) for 1 hour. The bacteria were washed, fixed and stained with DAPI (blue). Images were taken using confocal microscopy.

**Figure 4:**
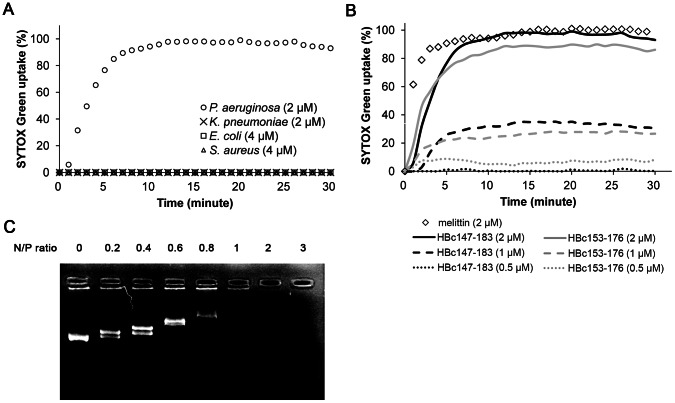
Possible bactericidal mechanism of HBc147-183. **(A) SYTOX Green uptake of ***P. aeruginosa* (circle), *K. pneumoniae* (cross) *E. coli* (square), and *S. aureus* (triangle) by HBc147-183.**** Measurements of the fluorescence were recorded every minute. (B) Dose-dependent curves of membrane permeabilization of *P. aeruginosa* by HBc147-183 (black) and HBc153-176 (grey) at 0.5, 1 and 2 µM (dots, dashes and solid line). 2 µM melittin (diamond) was used as a positive control. Samples were measured in triplicates. (C) DNA-binding activity of HBc147-183. HBc147-183 was mixed with pSUPER plasmid DNA at indicated N/P ratio for 30 minutes. The mobility of DNA was determined by gel retardation assay.

As shown in Figure 3, *P. aeruginosa*, *E. coli* and *S. aureus* were treated with FITC-labeled HBc147-183 corresponding to 0.5×MBC, and the localization of HBc147-183 was visualized using confocal fluorescence microscopy. The results showed that, upon peptide treatment, *P. aeruginosa*, *K. pneumonia* and *E. coli* appeared as hollow rods with fluorescence clearly defined bacteria surface, suggesting that HBc147-183 was accumulated on the membrane (Figure 3A–D). To understand better the effect of HBc peptides on the membranes, SYTOX Green uptake assay was performed. As shown in Figure 4A, a significant degree of membrane permeabilization was induced on *P. aeruginosa* upon the addition of 2 µM HBc147-183. Although it was also accumulated on the membrane of *K. pneumonia* and *E. coli*, 4 µM HBc147-183 was not able to induce membrane permeabilization as observed on *S. aureus*. Consistent with the bactericidal activity of HBc147-183 against *P. aeruginosa*, HBc153-176 caused the same membrane permeabilization within 10 minutes in a dose-dependent manner (Figure 4B). The results indicated that the bactericidal effect of HBc peptides on *P. aeruginosa* is directly through the membrane permeabilization with a fast kinetics similar to that of killing kinetics (Figure 2). On the other hand, HBc147-183 was found to penetrate through the membrane of *S. aureus* and localized in the cytoplasm (Figure 3E–G). In order to investigate the potential interaction between HBc 147-183 and DNA, HBc147-183 was mixed with pSUPER plasmid DNA at different N/P ratio (Materials and Methods) and analyzed by gel electrophoresis (Figure 4C). The results showed that the mobility of DNA was decreased when the ratio of peptide/DNA increased and the plasmid DNA was completely retarded at the ratio of 1, suggesting that HBc147-183 has a strong binding activity to plasmid DNA. Overall, it suggests that the bactericidal mechanisms of HBc147-183 on Gram-positive and Gram-negative bacteria may be completely different (see Discussion for further detail).

### Direct binding of HBc147-183 to LPS

**Figure 5:**
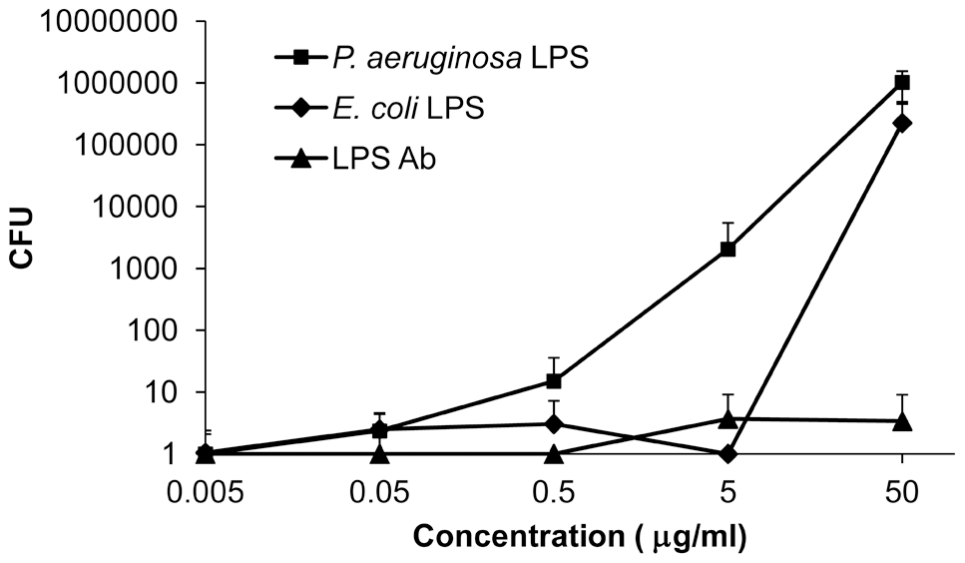
Dose response effects of LPS and LPS antibody (triangle) on the bactericidal activity of HBc147-183. LPS from *P. aeruginosa* (square) and *E. coli* (diamond), and LPS antibody (triangle) were mixed with *P. aeruginosa* and HBc147-183 (1×MBC) for 3 hours. The bacteria were then plated on MH agar for the measurement of viability. Samples were measured in triplicates.

To determine whether LPS of Gram-negative bacteria could serve as a potential target of HBc147-183, LPS (0.05 to 50 µg/ml) from either *P. aeruginosa* or *E. coli* (Sigma) were incubated with both *P. aeruginosa* and 2 µM HBc147-183 for three hours, respectively. The results showed that the bactericidal activity of HBc147-183 was significantly reduced by the addition of either LPS at the concentration of 50 µg/ml (Figure 5). In addition, HBc147-183 preferentially bound to the LPS from *P. aeruginosa*, rather than that from *E. coli*. However, the addition of anti-LPS antibody (Genetex Co.) cannot sufficiently neutralize the bactericidal activity of HBc147-183 (Figure 5). Taken together, it suggests that HBc147-183 could bind to not only LPS but also other target molecules on the membrane. Alternatively, HBc147-183 and the anti-LPS polyclonal antibody used here could bind predominantly to two different epitopes on the LPS.

**Figure 6:**
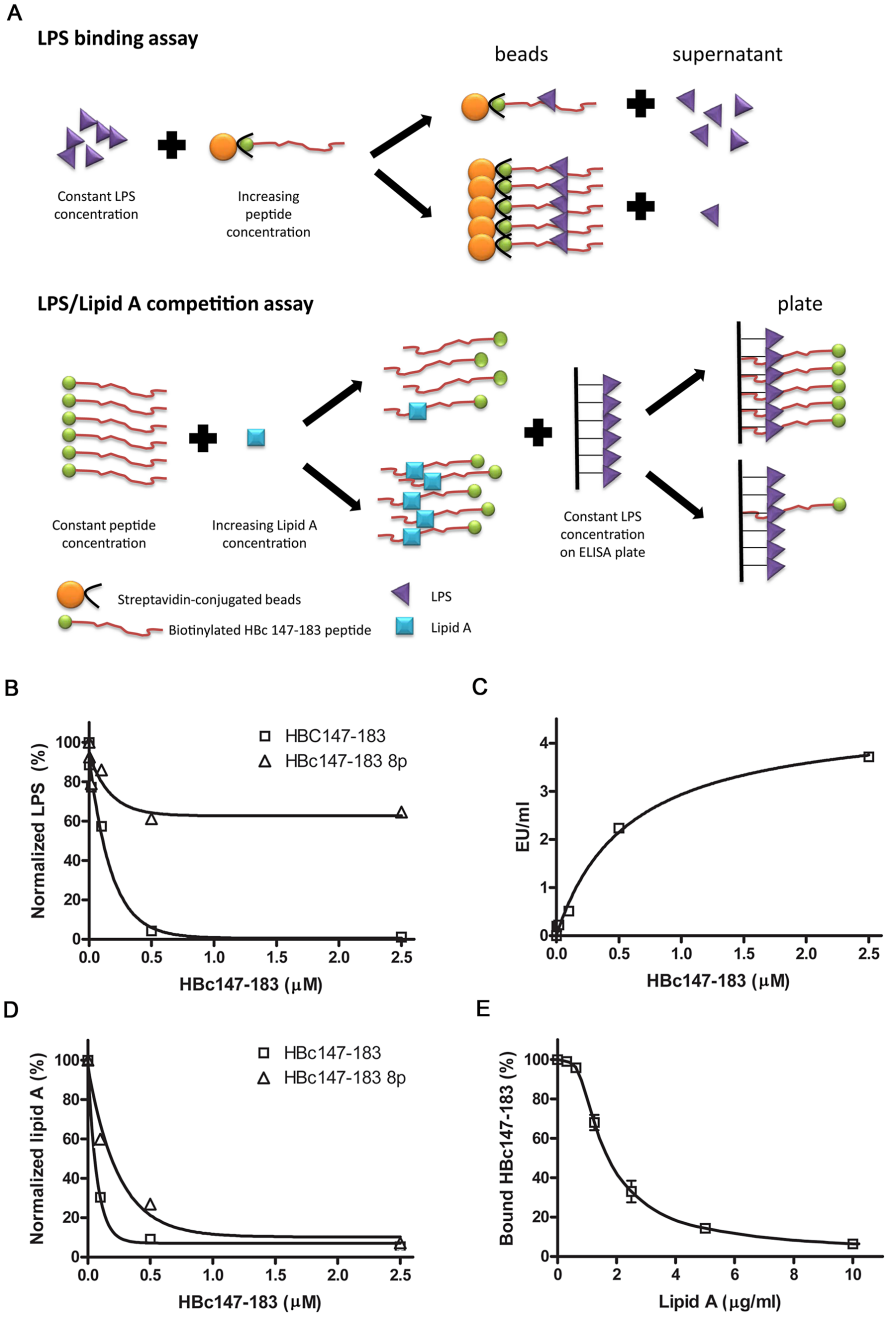
The ARD peptide HBc147-183 was shown to be capable of binding to LPS and Lipid A in several different *in vitro* binding assays. For each assay, samples were always measured in triplicates. (A) The cartoon illustrates the *in vitro* assays of peptide-LPS and peptide-Lipid A binding as well as LPS/Lipid A competition. (B) Constant amount of LPS was incubated with increasing concentrations of biotinylated ARD HBc 147-183 peptide on the streptavidine-conjugated beads (0, 0.004, 0.02, 0.1, 0.5 and 2.5 µM). Unbound LPS in the supernatant was measured with the LAL ELISA assay (Materials and Methods). The EU values were normalized with a control without peptide treatment. HBc147-183 8p (containing 8 phosphorylated amino acids) was also included as a control peptide due to its poor binding with LPS. (C) Beads-bound LPS was released into the supernatant by overnight digestion with trypsin agarose. Free LPS in the supernatant was analyzed with the LAL ELISA assay. Released LPS in the supernatant appeared to be in proportion to the amount of ARD peptide HBc147-183 on the beads. (D) Constant amount of Lipid A was incubated with increasing concentrations of HBc147-183 and HBc147-183 8P, respectively. The supernatant was also detected with LAL ELISA reagent. The result here is consistent with the notion that Lipid A can bind to HBc147-183 directly. (E) LPS/Lipid A competition assay. Constant amount of LPS (1 µg) was coated on each well on the ELISA plate, and then incubated with a reaction mixture containing constant amount of 10 nM HBc147-183 and increasing concentrations of Lipid A. The gradual increase of Lipid A reduced the amount of plate-bound ARD peptide HBc147-183 in a dose dependent manner.

As shown in the cartoon illustration of Figure 6A, the potential interaction between HBc147-183 and LPS (or Lipid A moiety) *in vitro* was investigated using several different binding assays (Materials and Methods). In Figure 6B, when increasing amount of HBc147-183 was bound to the strepavidine-conjugated Dynabeads and allowed incubation with constant amount of LPS, gradually increasing amount of LPS appeared to be depleted from the supernatant. HBc147-183 8p, which has eight ser/thr phosphorylations, was used in parallel as a control peptide. Similar results were obtained by another LPS testing method: Endosafe-PTS Cartridges (Charles River Laboratories). In Figure 6C, the beads-captured LPS were dissociated from the beads by trypsin agarose digestion of the ARD peptide HBc147-183. The amount of released LPS was measured by the LAL test (Materials and Methods). LPS contains mainly the polysaccharide and Lipid A moieties. To determine whether the ARD peptide can bind to Lipid A directly, we tested in Figure 6D the binding between Lipid A and the ARD peptide in a manner similar to Figure 6B. As expected, increasing amounts of HBc147-183 on the beads led to decreasing amounts of Lipid A remaining in the supernatant. The inverse correlation between the ARD peptide on the beads and the Lipid A in the supernatant (Figure 6D) is strikingly similar to what was observed previously between the ARD peptide HBc147-183 on the beads and LPS in the supernatant (Figure 6B). To directly demonstrate that the ARD peptide can bind to the Lipid A moiety of LPS, we performed a competition experiment between Lipid A and LPS (Figure 6E). The LPS-coated ELISA plates were incubated with constant amount of HBc147-183 (10 nM), which was premixed with varying concentrations of *E. coli* Lipid A (0 to 10 µg/ml). After extensive washing, plate-bound (i.e., LPS-bound) biotinylated peptide HBc147-183 was measured by streptavidin conjugated with HRP, followed by adding TMB substrates and color development (Materials and Methods). Indeed, the binding of HBc147-183 to LPS was significantly decreased by the increasing concentrations of Lipid A (Figure 6E). The result here lends support for the notion that Lipid A moiety of LPS can serve as a direct target for ARD peptide HBc147-183.

### Cytotoxicity

**Figure 7:**
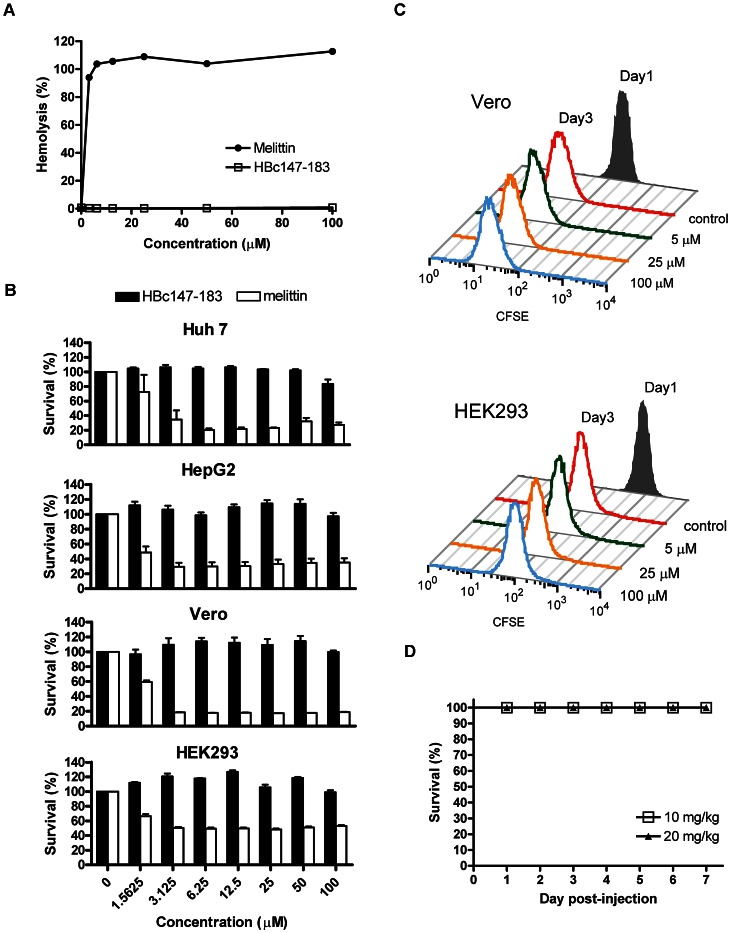
Cytotoxicity assays of ARD peptide HBc147-183. % human red blood cells (RBC). Compared to melittin, HBc147-183 showed no hemolytic activity. (B) Huh7, HepG2, Vero and HEK293 cells were incubated with varying concentrations (0 to 100 µM) of HBc147-183 (black) and melittin (white) for 1 hour at 37°C. The effects on cell viability were determined by MTT assay. Melittin was used as a positive control. HBc147-183 showed no detectable effect on cell viability, while melittin exhibited strong toxicity. (C) Kidney cells Vero and HEK293 were stained with CFSE and seeded at day 0 (Materials and Methods). At day 1, cells were incubated with varying concentrations (0 to 100 µM) of HBc147-183 for 1 hour. Cell proliferation at day 1 and day 3 were determined by flow cytometry. Similar to the mock control experiment, no significant effect on Vero and HEK293 cells was detected. Samples assayed in Figure 7A–C were always measured in triplicates. (D) *In vivo* toxicity of ARD peptide HBc147-183 was determined using three-week old male ICR mice. The mice were injected intraperitoneally with peptide (10 and 20 mg/kg of body weight). All mice were alive after 7 days.

To determine the cytotoxicity of HBc peptides, we measured the hemolytic activity of HBc147-183. Compared to the melittin control, no detectable hemolysis by HBc147-183 was observed after one hour of incubation (Figure 7A). In addition, MTT assay was performed to determine the cytotoxicity of HBc147-183 to human hepatoma (Huh 7 and HepG2 cells) and kidney cells (Vero and HEK293 cells). The viability of cells treated with melittin at low dose (3.125 µM) was significantly decreased. In contrast, HBc147-183 caused only a low level of cytotoxicity at the concentration of 100 µM (Figure 7B). The CFSE cell proliferation assay was also performed to determine the effect of HBc147-183 on the proliferation of Vero and HEK293 kidney cells. In comparison to day 1, CFSE intensity of cells treated with HBc147-183 (5, 25 and 100 µM) decreased to the same level as the mock control on day 3 (Figure 7C), suggesting that ARD peptide HBc147-183 has no significant effect on cell proliferation.

### Animal model

**Figure 8:**
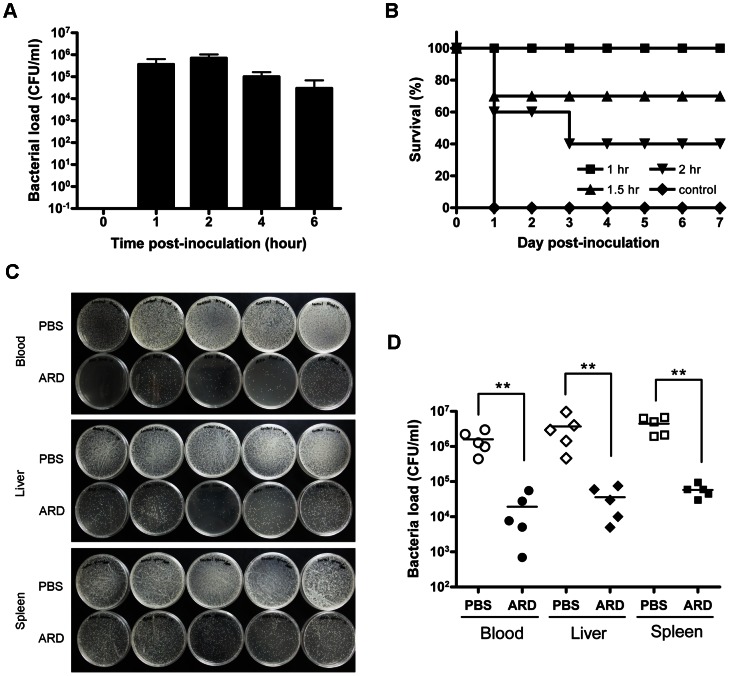
*In vivo* studies of the protection activity of ARD peptide HBc147-183 against *S. aureus*. (A) Three-week old male ICR mice were challenged with a lethal dose of *S. aureus* ATCC 19636 and then divided into five separate groups for five different time points. At each indicated time point (n = 5), blood samples were collected, diluted and plated on BHI agar. The number of bacteria was counted the following day. A maximal bacterial load in the blood was observed at 2 h post-inoculation. The data were shown in mean ± SD. (B) ICR mice inoculated with a lethal dose of *S. aureus* as described above were treated by intraperitoneal injection with ARD peptide (10 mg/kg) at 1, 1.5 or 2 h post-inoculation, respectively. Each group contained 10 mice. All mice (100%) treated with the PBS control died at day 1, while treatment of ARD peptide at 1, 1.5 or 2 h post-inoculation protected the mice with survival rates of 100%, 70% and 40% after 7 days, respectively. (C) As described above, ICR mice were i.p. inoculated with *S. aureus*, followed by i.p. injection with PBS (n = 5) or 10 mg/kg ARD peptide (n = 5) at 1 h post-inoculation. At 4 h post-inoculation, blood, liver and spleen were collected. Liver and spleen samples were homogenized, diluted and, together with blood samples, plated on BHI agar. The number of bacteria was counted the following day. In comparison to mice treated with PBS, treatment of ARD peptide effectively reduced the bacterial load in blood, liver and spleen. (D) Quantitative comparison of bacterial loads in blood, liver and spleen samples of mice treated with PBS (open circle, diamond and square) versus ARD peptide HBc147-183 (solid circle, diamond and square). The line indicated the mean of bacterial load. **P<0.01 (Mann-Whitney U test) for PBS and ARD peptide HBc147-183.

**Figure 9:**
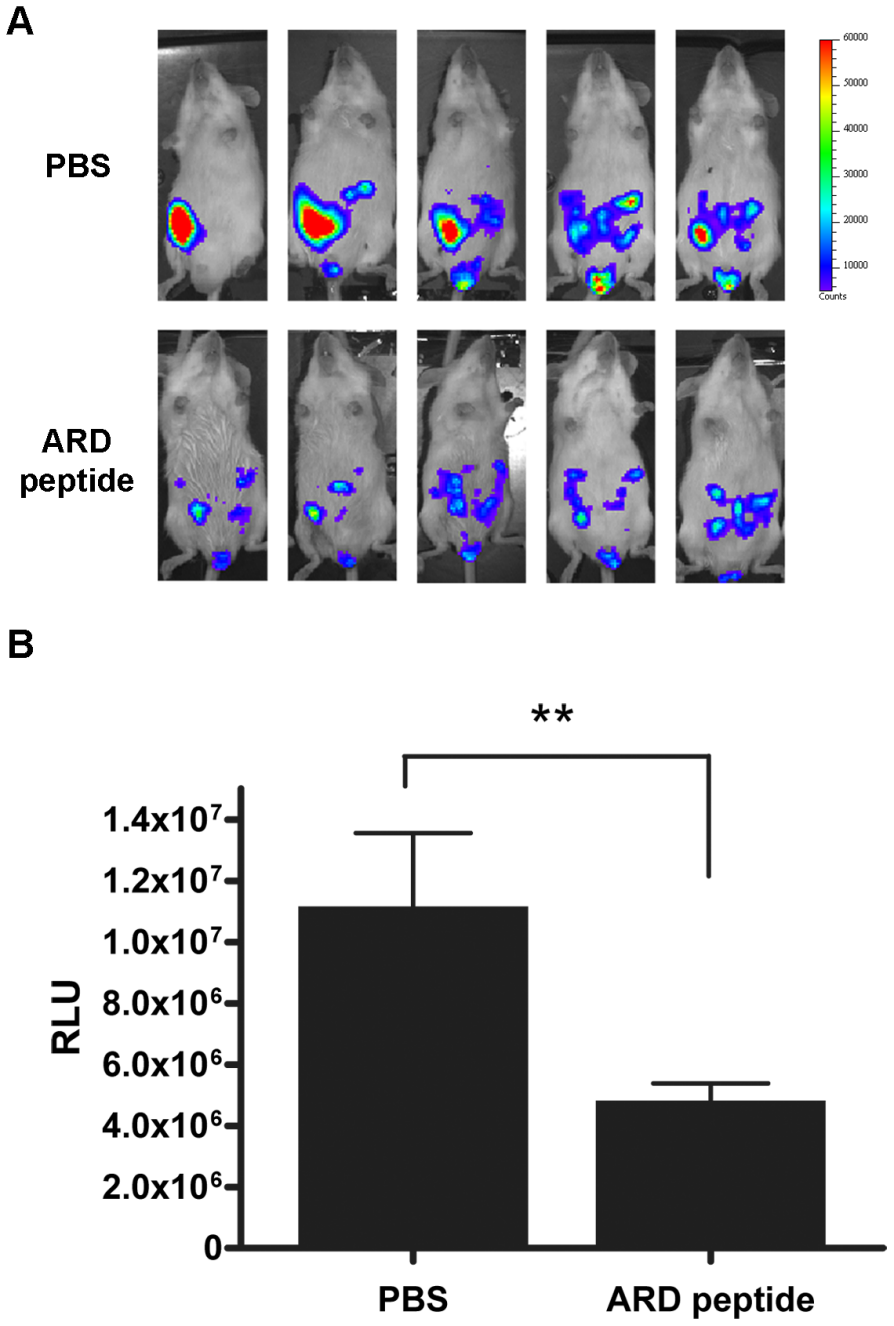
IVIS analysis of *in vivo* antimicrobial activity of ARD peptide against K. pneumoniae. (A) *K. pneumoniae*-infected mice were treated with either PBS (n = 5) or 10 mg/kg ARD peptide (n = 5) at 1 hour post-inoculation. Four hours post-inoculation, mice were anesthetized and imaged. Bacterial load was displayed in the photographic image with an overlay of bioluminescence. False color imaging represents intense luminescence in red, moderate luminescence in green and low luminescence in blue and purple. (B) Total flux was quantified by IVIS imaging software. **P<0.01 (Mann-Whitney U test) for PBS and ARD peptide HBc147-183.

To conduct the experimental infection with bacteria, we i.p. inoculated mice with *Staphylococcus aureus* ATCC 19636 (4×10^6^ cfu/mouse). Bacterial load in blood at 1, 2, 4 and 6 hours post-inoculation was determined. As shown in Figure 8A, bacteria rapidly transferred to the blood compartment from peritoneal cavity. Within 2 hours, the number of bacteria in the blood achieved the maximum (10^6^ cfu/ml). Thereafter, the number of bacteria in the blood gradually decreased spontaneously. To distinguish the ARD peptide-mediated from the spontaneous clearance, we therefore tested the *in vivo* protection activity of the ARD peptide within 2 hours post-inoculation. Briefly, mice were i.p. inoculated with *Staphylococcus aureus* ATCC 19636 and received a single dose of 10 ml/kg PBS or a single dose of 10 mg/kg ARD peptide at 1, 1.5, 2 hours post-inoculation, respectively. Mice (n = 10) treated with PBS died within 24 hours post-inoculation (Figure 8B). In contrast, administration of ARD peptide (10 mg/kg) at 1 hour post-inoculation can effectively protect all mice (n = 10) from death at day 7. When we administered ARD peptide at 1.5 (n = 10) and 2 (n = 10) hours post- inoculation, survival rates were decreased to 70% and 40%, respectively. Instead of using death as a surrogate indicator of the antimicrobial activity of ARD peptide, we also determined directly the *in vivo* effect of ARD peptide on bacterial load of infected mice (Figure 8C). Mice were inoculated with *Staphylococcus aureus* as before and treated with 10 ml/kg PBS (n = 5) or 10 mg/kg ARD peptide (n = 5) at 1 hour post-inoculation. Four hours post-inoculation, bacterial load in blood, liver and spleen samples of control mice were in the range of 10^6^ cfu/ml (Figure 8C and 8D). Administration of ARD peptide significantly reduced the bacterial load (∼10^4^ cfu/ml) by 100-fold in blood, liver and spleen than the PBS control mice (*P*<0.01). In addition to *Staphylococcus aureus*, we also examined the *in vivo* antimicrobial activity of ARD peptide on *K. pneumoniae* using an IVIS imaging system. Similar to the change in bacterial load of *S. aureus*, bioluminescence of mice inoculated with *K. pneumoniae* Xen39 peaked at 2 hour post-inoculation (data not shown). We then treated *K. pneumoniae* Xen39-infected mice with PBS or ARD peptide at 1 hour post-inoculation, respectively. The results showed that the bioluminescence of ARD peptide-treated mice was very weak, whereas PBS control showed a more extensive bioluminescence (Figure 9A). There was a significant difference in the overall RLU values of mice treated with PBS versus ARD peptide (*P*<0.01) (Figure 9B). Taken together, the results indicated that HBc147-183 exhibited significant antimicrobial activity *in vivo*.

## Discussion

In this study, we present a novel antimicrobial peptide (HBc147-183) isolated from the C-termial domain of HBc. The computer program, based on the antimicrobial peptide database [37], predicted unfavorably that HBc147-183 could serve as an antibacterial peptide, due to its very low content of hydrophobic amino acids. In contrast to the computer prediction, surprisingly, HBc147-183 exhibited a broad-spectrum antimicrobial activity.

### Drug resistance to polymyxin and colistin

Many clinical isolates of *P. aeruginosa*, *K. pneumonia*, *A. baumannii* and *S. aureus* are highly pathogenic and are resistant to aminoglycosides, beta-lactams, and fluoroquinolones [38], [39], posing a serious threat to human health. In the past decades, polymyxins, such as polymyxin B and colistin (polymyxin E), were considered the “Last Hope” antibiotics, and were increasingly used in clinical settings to treat multidrug resistant bacteria [40], [41]. However, polymyxin-resistant bacteria have also emerged most recently, and the studies of adaptive resistance to polymyxin have been reported [36], [42]. In Table 2, we tested the bactericidal activity of HBc147-183 to colistin-resistant *P. aeruginosa* and *A. baumannii*. While colistin-resistant *P. aeruginosa* exhibited cross-resistance to ARD peptide HBC147-183, we found a strong activity of HBc147-183 (MBC = 0.5–1 µM) against all tested colistin-resistant *A. baumannii*. Therefore, it appears that *A. baumannii* and *P. aeruginosa* may have adopted different strategies to acquire resistance to colistin. It would be interesting to investigate further in the future whether the so-called two component regulatory systems, such as ParR-ParS and CprR-CprS, could contribute to the colistin-induced cross-resistance to the HBc ARD peptide [41], [42], [43]. It has been proposed that Lipid A modification could be responsible, at least in part, for the resistance to polymyxin in *P. aeruginosa* [43]. In this regard, it is noteworthy that our ARD peptide could bind to Lipid A of *E. coli* and LPS of *P. aeruginosa* (Figure 6). It might be relevant to compare the lipid A structures between colistin-resistant *P. aeruginosa* and *A. baumannii* in the future (Table 2). Most importantly, our studies here open up the possibility that ARD peptide could be used for treatment of colistin- resistant *A. baumannii* in the future.

### Critical sequences of HBc ARD peptide for its bactericidal activity

As shown in Table 1, the bactericidal activity of phosphorylated peptides and mutant peptide (Arg to Ala) were significantly reduced. In addition, a highly phosphorylated peptide (HBc147-183 8p) showed significantly reduced binding activity to LPS compared to non-phosphorylated HBc147-183 (Figure 6B and 6D). It suggested that arginine residues and positive charge are very important for the activity. While only the full-length HBc147-183 (ARD I–IV) was effective against the tested Gram-positive bacteria, *S. aureus*, ARD II–IV (HBc153-176) and ARD I–III (HBc147-167), in a less than full-length context, exhibited strong activity against Gram-negative *P. aeruginosa* and *K. pneumoniae*, respectively (but not *E. coli*). Consistently, phosphorylation of S162 and S170 resulted in much weaker activity against *P. aeruginosa* and *K. pneumoniae*, while phosphorylation at several other positions showed no apparent attenuation effects (Table 1). In summary, a minimal amount of both arginines and positive charge of HBc ARD peptides appeared to be important for effective bactericidal activity against these different Gram-positive and Gram-negative bacteria.

### Comparisons with other AMPs

It is surprising that the ARD domain of HBc protein (HBc147-183) exhibits novel and broad spectrum antimicrobial activity. This potent peptide shares some degree of similarity with several antimicrobial peptides in literature, such as protamine (PRRRRSSSRPVRRRRRPRVSRRRRRRGGRRRR) [29] and Drosocin (GKPRPYSPRPTSHPRPIRV) [44]. Protamine is a polycationic peptide found in the nuclei of sperm of different animal species [29]. It consists of four arginine clusters. Radial diffusion assay has shown that a single arginine-rich domain (RRRR) is sufficient for antimicrobial activity, especially against Gram-negative bacteria [16]. Unlike protamine, the arginine-rich domain of HBc147-183, such as ARD I–II and ARD III–IV, were not sufficient for the antimicrobial activity. In addition, sequence alignment by anitimicrobial peptide database revealed that HBc153-176 shares 44% amino acid sequence homology with Drosocin, which is a proline-rich peptide isolated from Drosophila. However, except for *P. aeruginosa*, Drosocin is predominately active against most Gram-negative bacteria. Drosocin kills bacteria via an apparently non-membranolytic mechanism [44], [45]. Taken together, HBc ARD is a novel peptide with a broad spectrum bactericidal activity quite distinct from the other known arginine-rich antimicrobial peptides.

### Bactericidal mechanisms

AMPs can act by several mechanisms, including permeabilization of the membrane, as well as inhibition of the synthesis of protein, DNA, or cell wall [12]. Gram-negative bacteria have been shown to contain receptors for antimicrobial peptides, such as lipopolysaccharide [46], and membrane proteins, such as OprI and Lpp [47], [48]. Our results here showed the membrane localization of HBc147-183 on Gram-negative bacteria (Figure 3) and the neutralization activity of LPS from either *P. aeruginosa* or *E. coli* (Figure 5). It suggests that HBc147-183 could have a strong binding activity to LPS. Indeed, based on several *in vitro* binding assays, our results revealed a direct binding of HBc147-183 to LPS and Lipid A (Figure 6B, 6C and 6D). Furthermore, Lipid A moiety of LPS was shown to be one major direct target of HBc147-183 (Figure 6E). However, incubation of LPS antibody with *P. aeruginosa* and HBc147-183 failed to neutralize the bactericidal activity of HBc ARD peptide (Figure 5). One interpretation for this negative result, among several others, is that HBc147-183 could bind not only LPS but also some other molecules on the bacterial membrane.

Previous studies have shown that the LPS of *P. aeruginosa* and *E. coli* have diverse structures of lipid A, a core component of LPS [49], [50]. Indeed, we found a better neutralization effect of the LPS from *P. aeruginosa* than that from *E. coli* (Figure 5). The preference of binding by HBc147-183 for the LPS of *P. aeruginosa* is correlated with its stronger bactericidal activity against *P. aeruginosa*.

Previously, it has been shown that AMPs causing membrane permeabilization exhibited fast killing kinetics, while AMPs and antibiotics targeting intracellular components exhibited slow killing kinetics [51]. The mode of action of HBc147-183 on *P. aeruginosa* could be related to membrane permeabilization based on the fast killing kinetics (Figure 2) and its membrane localization (Figure 3A and 3B). This speculation is also supported by the results of SYTOX Green uptake experiment (Figure 4A). Like *P. aeruginosa*, HBc147-183 was also accumulated on the membrane of *K. pneumonia* and *E. coli*. However, the killing kinetics and SYTOX Green uptake experiments of *K. pneumonia* and *E. coli* did not support for a mechanism of membrane permeabilization. Bactericidal mechanisms other than membrane permeabilization can be cited. For example, mammalian peptidoglycan recognition protein (PGRP) has been reported to kill bacteria by activating protein sensing two-component systems [52]. It remains to be further investigated how bacteria can be killed by the ARD peptides using a mechanism other than membrane permeabilization.

In the case of Gram-positive bacteria, we found that HBc147-183 was not accumulated on the membrane (Figure 3). Instead, it can enter the cytoplasm of *S. aureus* without any apparent development of membrane permeabilization (Figure 4A). In addition to LPS, HBc147-183 can also bind strongly to plasmid DNA (Figure 4C). Taken together, the bactericidal mechanism of HBc147-183 against Gram-positive bacteria appeared to be more similar to Buforin II, which was reported to kill bacteria by binding to DNA and RNA after penetrating bacterial membrane [53].

Other arginine-rich peptides, such as Penetratin [54], Tat peptide [55], and oligoarginine [56], have been reported to be able to enter the mammalian cells. Although HBc147-183 can penetrate through the cell membrane of Huh 7 and HepG2 cells (data not shown), we observed no significant cytotoxic effect on human hepatoma cells Huh 7 and HepG2, as well as kidney cells Vero and HEK293, even at high peptide concentration (100 µM) by MTT assay (Figure 7B) and proliferation assay (Figure 7C). Taken together with the results from the hemolytic assay (Figure 7A), HBc147-183 appears to be much safer relative to melittin in cell culture [57]. Indeed, in our animal model study, we observed no apparent *in vivo* toxicity of ARD peptide at 20 mg/kg dose in the ICR mice by i.p. injection (Figure 7D). In fact, at as low as 10 mg/kg level, treatment of ARD peptide can protect mice from death (Figure 8B). In contrast, all mice receiving the PBS control were dead soon after bacterial inoculation. In addition to the sepsis survival model, treatment of ARD peptide (10 mg/kg) also resulted in a significant reduction of bacterial load of *S. aureus* and *K. pneumoniae*, whereas PBS control mice showed high levels of bacterial load (Figure 8C–D and Figure 9). The results demonstrated the *in vivo* antimicrobial potency of HBc ARD peptide.

#### Intestinal microbiota and establishment of chronic HBV infection

It has been noted that there might be an intriguing relationship between the development of the immune system and the flora of the gastrointestinal tract [58], [59], [60]. The composition of the commensal flora, or even a single species of microorganism, could play an important role in shaping the balance of specific T cell subsets [61], [62], [63], [64]. Intestine and liver are known to communicate in an intimate manner through the portal vein. In addition, increased bacterial translocation of enteric organisms is common in patients with chronic liver diseases [65], [66], [67]. It is therefore tempting to speculate that the anti-microbial activity of HBc ARD could contribute its influence on the commensal signals from the GI tract, particularly in the newborns, leading to the establishment of immune tolerance in the liver. Coincidentally, the C-terminus of the ARD domain of HBc was recently reported to play an important role in the persistence of HBV in a mouse model [68]. It will be interesting to investigate further in the future whether the anti-microbial activity of HBc ARD could contribute to the intrahepatic immune tolerance in HBV-infected newborns.

In conclusion, we report here that HBc peptides can exhibit a broad-spectrum activity against bacteria. It is anticipated that HBc ARD peptide might have the potential to serve as a novel antimicrobial agent, by itself or in combination with other antibiotics, in the future.
